# The All Our Babies pregnancy cohort: design, methods, and participant characteristics

**DOI:** 10.1186/1471-2393-13-S1-S2

**Published:** 2013-01-31

**Authors:** Sheila W McDonald, Andrew W Lyon, Karen M Benzies, Deborah A McNeil, Stephen J Lye, Siobhan M Dolan, Craig E Pennell, Alan D Bocking, Suzanne C Tough

**Affiliations:** 1 Department of Paediatrics, University of Calgary, Calgary, AB, Canada; 2 Department of Pathology and Laboratory Medicine, University of Saskatchewan, Saskatoon, SK, Canada; 3 Faculty of Nursing, University of Calgary, Calgary, AB, Canada; 4 Department of Population and Public Health, Alberta Health Services, Calgary, AB, Canada; 5 Department of Obstetrics and Gynecology, University of Toronto, Toronto, ON, Canada; 6 Department of Obstetrics and Gynecology and Women’s Health, Albert Einstein College of Medicine of Yeshiva University, Bronx, NY, USA; 7 School of Women’s and Infants’ Health, University of Western Australia, Perth, Western Australia, Australia; 8 Department of Obstetrics and Gynecology, University of Toronto, Toronto, ON, Canada; 9 Department of Community Health Sciences, university of Calgary, Calgary, AB, Canada

## Abstract

**Background:**

The prospective cohort study design is ideal for examining diseases of public health importance, as its inherent temporal nature renders it advantageous for studying early life influences on health outcomes and research questions of aetiological significance. This paper will describe the development and characteristics of the All Our Babies (AOB) study, a prospective pregnancy cohort in Calgary, Alberta, Canada designed to examine determinants of maternal, infant, and child outcomes and identify barriers and facilitators in health care utilization.

**Methods:**

Women were recruited from health care offices, communities, and through Calgary Laboratory Services before 25 weeks gestation from May 2008 to December 2010. Participants completed two questionnaires during pregnancy, a third at 4 months postpartum, and are currently being followed-up with questionnaires at 12, 24, and 36 months. Data was collected on pregnancy history, demographics, lifestyle, health care utilization, physical and mental health, parenting, and child developmental outcomes and milestones. In addition, biological/serological and genetic markers can be extracted from collected maternal and cord blood samples.

**Results:**

A total of 4011 pregnant women were eligible for recruitment into the AOB study. Of this, 3388 women completed at least one survey. The majority of participants were less than 35 years of age, Caucasian, Canadian born, married or in a common-law relationship, well-educated, and reported household incomes above the Calgary median. Women who discontinued after the first survey (n=123) were typically younger, non-Caucasian, foreign-born, had lower education and household income levels, were less likely to be married or in a common-law relationship, and had poor psychosocial health in early pregnancy. In general, AOB participants reflect the pregnant and parenting population at local and provincial levels, and perinatal indicators from the study are comparable to perinatal surveillance data.

**Conclusions:**

The extensive and rich data collected in the AOB cohort provides the opportunity to answer complex questions about the relationships between biology, early experiences, and developmental outcomes. This cohort will contribute to the understanding of the biologic mechanisms and social/environmental pathways underlying associations between early and later life outcomes, gene-environment interactions, and developmental trajectories among children.

## Background

Population-based cohort studies are important sources of data to investigate life course processes and to identify aetiological determinants of health and disease outcomes in later life [1]. As they are not specific to a diseased population, they provide insight on what constitutes typical trajectories and minor variations within the normal range of development. Pregnancy and birth cohort studies are particularly salient for studying early origins of health and disease that begin in fetal life and infancy. Indeed, the causal underpinnings of many common diseases in adulthood (e.g., cardiovascular disease, obesity, psychopathology) have roots *in utero* and the early postnatal phase [2-8]. Early identification of threats to well-being is important for the development of preventive and early intervention strategies to optimize health and health care for individuals and communities. Cohort studies can provide important aetiological, descriptive and surveillance information about early risk factors for disease that can inform research, policy, programs, and practice.

Advantages of cohort studies for examining development and links between early and later life outcomes are well established [9-11]. The prospective cohort study design is especially suited for examining associations that require consideration of temporality and are less subject to recall bias and reverse-causality bias compared to other epidemiological study designs [1,9]. An important strength of longitudinal studies is their potential for investigating trajectories of development and identifying sensitive periods of risk or resilience [9,12]. Furthermore, in longitudinal research, there is a higher probability of discovering true exposure outcome relationships (i.e., causal relationships) when one exists [12]. An additional advantage relates to efficiency gained through the breadth of data collection and ability to assess a range of possible causes and outcome variables, although in cases of rare but important outcomes, collaboration with similar studies, or a more suitable design (i.e., case-control) is warranted [9].

The prospective cohort study has emerged as an important study design to investigate gene-environment interactions in diseases of major public health importance [1]. Although the case-control study remains a widely used method for examining genetic and environmental determinants of complex disease, they are subject to significant sources of bias that relate to subject selection and measurement of exposures and outcomes [1]. Prospective cohort studies and their substudies (e.g., nested case-control studies) can address some of these irremediable sources of bias and offer complementary and innovative sources of information for studying early origins of later disease and gene-environment interactions. A number of prospective pregnancy and birth cohorts studies exist in both developing and developed countries, and many have contributed to understanding the role of the pre- and postnatal environment on later life health, crucial for aetiological and prevention research; examples include European cohorts such as The Avon Longitudinal Study of Parents and Children (ALSPAC) [13], the Generation-R study [14], the Danish National Birth Cohort study [15], the Millennium Cohort Study [16], and North American cohorts such as the National Children’s Study [17], and the Ottawa and Kingston Birth Cohort [18]. This paper will describe the development and characteristics of the All Our Babies (AOB) study, a prospective pregnancy cohort study in Calgary, Alberta, Canada.

## Methods

### Overview

The AOB study (n=3388) was designed to examine maternal and infant outcomes during the perinatal period and to identify current barriers and facilitators to accessing health care services in Calgary, Alberta. A further objective that was incorporated approximately one year after the start of recruitment was to examine biological and environmental determinants of adverse birth outcomes, specifically spontaneous preterm birth, for which approximately half of the AOB sample (n=1862) provided blood samples at two time points during pregnancy, and cord blood, when retrievable, was collected at birth (n=1399). The biological data collection and storage provides whole blood, plasma, and serum samples from which lymphocytes, cytokines, and proteins may be isolated and RNA and DNA will be extracted for micro-array analysis and future measurement. Cord blood samples will be used for future studies. Biological data collection methodology has been previously described [19]. Currently, the AOB study is collecting observational data beyond the perinatal period at 12 months, 24 months, and 36 months. Future data collections at key developmental time points are planned. Overall recruitment of the AOB cohort as well as observational data collection procedures during the perinatal period and early childhood are described in turn below.

### Ethical approval

This study was approved by the Child Health Research Office and the Conjoint Health Research Ethics Board of the Faculties of Medicine, Nursing, and Kinesiology, University of Calgary, and the Affiliated Teaching Institutions (Ethics ID 20821 and 22821). Participants provided consent at the time of recruitment and were provided copies of the consent form for their records.

### Recruitment

**Figure 1:**
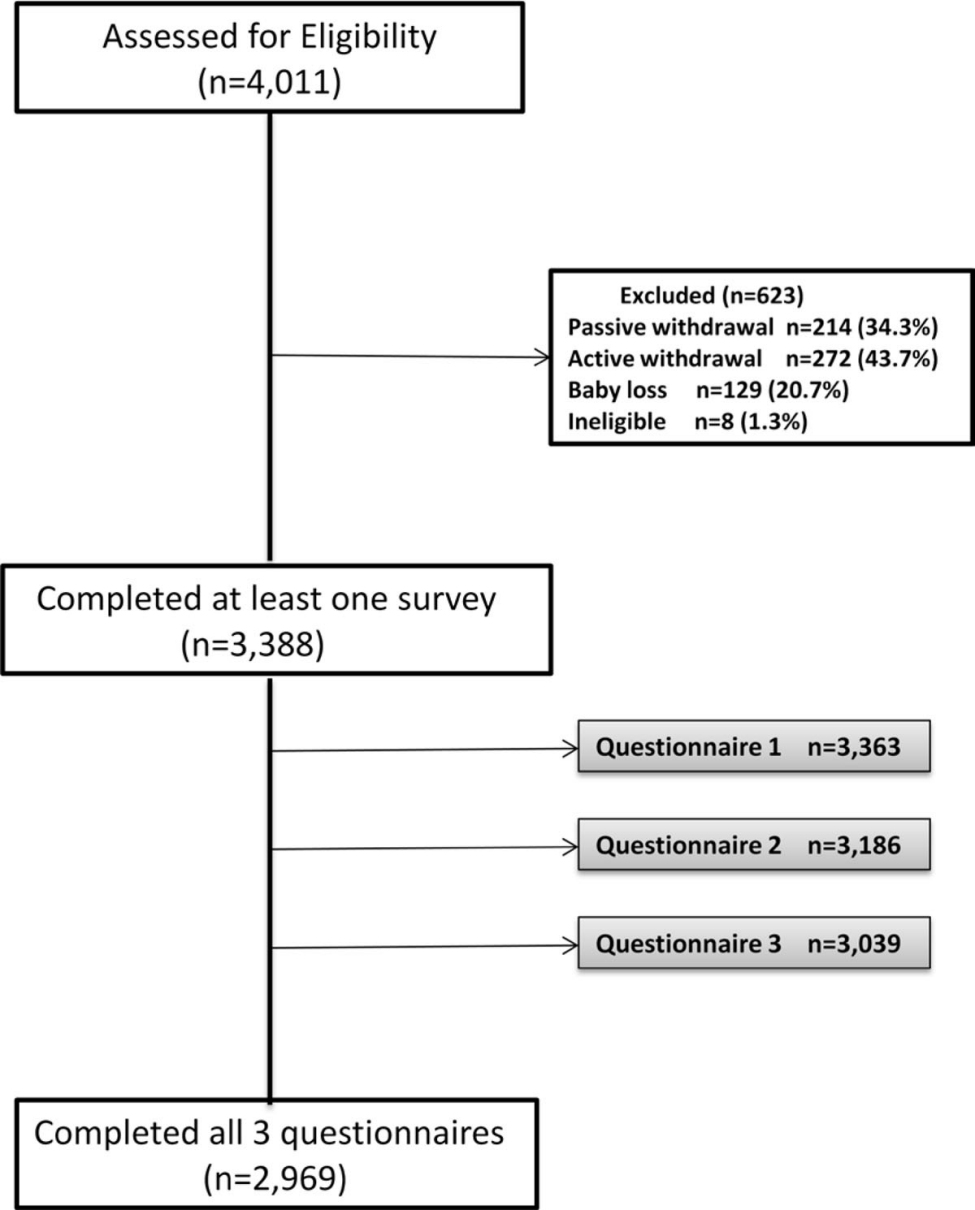
AOB participant recruitment

A planned approximate 3-year recruitment strategy for the AOB study began in May, 2008 and was completed in December, 2010. A total of 4011 pregnant women were assessed for eligibility from primary health care offices (n=573), community posters and word of mouth (n=675), and through a city-wide single provider public health laboratory service (Calgary Laboratory Services; n=2763) (Figure 1). The AOB cohort is population-based and the largest proportion of recruited participants (69%) was collected through Calgary Laboratory Services. Women were eligible if they were less than 24 weeks and 6 days gestation age at the time of recruitment, at least 18 years of age, receiving prenatal care in Calgary, and able to complete the questionnaires in English. Eight women were deemed ineligible at time of recruitment due to a language barrier. The most common reason for discontinuation from the study was active method of withdrawal (44%), including but not limited to: loss of interest, lack of time, reasons related to blood collections or linkage to medical records (although participants were not obligated to provide consent for these processes to participate), and lack of partner support. Passive withdrawals (34%) included geographical moves, lost to follow-up, or unknown reasons, while ineligible (1%) included those who self-defined as English as a Second Language, as noted above. Baby losses (21%) included both miscarriages and neonatal/infant loss (Figure 1).

### Data collection (perinatal period)

Eligible participants (n=4003) were invited to complete three questionnaires at separate time points across the perinatal period and 85% completed at least one questionnaire (Figure 1). In the AOB study, both cross-sectional and longitudinal responses contribute valuable information, with response rates ranging from 76% to 84% across the three data collection time points (Figure 1). Seventy-four percent completed all three questionnaires (Figure 1). These comprehensive questionnaires took about 25 minutes each to complete and included questions about the participant’s pregnancy history, health service utilization, demographics, lifestyle, mental, psychosocial and physical health, life events, quality of life, work status, parenting morale, and breastfeeding (see additional file 1 for a description of variables assessed in the AOB study by data collection time point). The questionnaires were developed with input from health care providers, epidemiologists and community program experts. Standardized tools were included as part of the questionnaires when available, and questions were created specifically for the study when standardized items or previously developed items were not suitable. The questionnaires were pilot tested on approximately 10-12 pregnant women in the community to ensure clarity and cultural sensitivity. Relevant resources, such as the Mental Health Help Line, were provided in the questionnaires where sensitive questions were asked. In addition to the questionnaires, all participants were asked to provide consent for the research team to access their prenatal and birth record data, including past pregnancy history, medical history and current conditions, pregnancy complications, labour/birth outcomes, and infant health data (Table 1).

**Table 1:** Information retrieved from hospital and medical records in the AOB study

Maternal history
Smoking, drug dependent
Pre-existing diabetes, heart disease, hypertension
Chronic renal disease, other chronic disorder, auto immune conditions
**Maternal past pregnancy history**
Previous term births, past preterm birth, previous preterm deliveries
Number of previous c-sections
Abortion, stillbirth(s), neonatal death, major congenital anomaly
History of intrauterine growth restriction, SGA, LGA

**Indicated pregnancy**
Smoking, drinking
Date admitted to labour and delivery
Assisted conception
Gravidity, parity
Maternal height <=152cm, maternal weight (<=45Kg, >=91Kg), poor weight gain
Antepartum risk score

**Pregnancy complications/problems**
Infection in pregnancy (GBS, HIV, HepB, other), fever, UTI
Poly/oligo, ROM <37 wks, bleeding
Pregnancy induced hypertension, gestational diabetes
Proteinuria, anemia
Cerclage, pre-eclampsia, eclampsia, abruption, prolonged premature rupture of membranes, placenta previa,
Intrauterine growth restriction, polyhydramnios, chorioamnionitis

**Delivery**
Site, type of delivery provider
Multiple pregnancy, maternal age at delivery, gestation, pregnancy >=41 weeks
Admitted for elect c-section, reason for operative delivery
Indication for induction, cervical dilatation at presentation, type of delivery, delivery mode
(Fetal) presentation in labour, trial of labour
Method of induction (oxytocin, artificial rupture of membranes, other)
Narcotics in labour, epidural in labour, Antenatal steroids, use of intrapartum antibiotics
Second stage (minutes), third stage (minutes)

**Fetal**
Neonatal gender, birth weight, date/time, disposition
5 minute Apgar score
Meconium, resuscitation
NICU admission, congenital anomaly

**Maternal**
Maternal discharge date, maternal discharge disposition, length of stay
Breastfeeding at discharge

The mailed questionnaire packages included an information letter, consent form, contact information form, questionnaire, and postage pre-paid return envelope. The participants were asked to complete the first questionnaire at recruitment (before 25 weeks gestation), the second between 34-36 weeks gestation, and the third at 4 months postpartum. The questionnaires were returned to the research team by regular post. Trained research assistants contacted the participants if data were missing or clarification of responses was required. Participants who failed to return their questionnaire within three weeks were contacted by telephone and/or e-mail and reminded to complete the questionnaire; multiple attempts were made until the participant was contacted and provided the opportunity for a repeat mail-out or to complete the questionnaire over the telephone. After completion and return of their questionnaires at each time point, the participants were provided with a token of appreciation such as library and grocery store gift cards. In order to keep participants engaged and updated, congratulation cards were sent after the birth of their baby, as well as newsletters semi-annually containing such information as project progress and findings (e.g., most popular baby names), preliminary results and research team member profiles.

All raw data was scanned into Teleform (Version 10.1) and went through a verification process to improve accuracy. Data was exported and cleaned according to data cleaning guidelines, including data coding, frequency editing, and cross-sectional and longitudinal logical editing [20]. Information across the three time points was linked according to a unique identifier that was assigned to each participant at study entry, preserving participant confidentiality. Information from medical charts was linked with questionnaire data by means of personal health numbers. Questionnaire and medical data were stored separately from participant data, the latter which include personal information such as name, address, and personal health number. This separation acts to set up a central barrier between administrative data needed for conducting the study and anonymised data needed to answer the research questions. Both hard copies and electronic copies of data are stored in a secure environment and adhere to security and confidentiality protocol as per the institutional ethics board and recommended guidelines [20].

### Data collection (early childhood)

For each follow-up data collection wave in early childhood (12 months, 24 months, and 36 months), the AOB study team developed a 20 page questionnaire to measure domains of maternal physical and mental health, parenting, health care utilization, and family well-being. Specific questions and standardized tools to assess child developmental outcomes and milestones were also administered. In order to understand trajectories of development, the same construct (e.g., maternal depression) was assessed across time, using the same tool if appropriate. Furthermore, relevant domains of functioning at each time point were assessed. For example, questions regarding work-life balance/return to work and separation anxiety were asked at the 12 month data collection time point, and questions regarding child behaviour and oral health were deemed important for the 36 month follow-up. Outcomes of interest that will be measured in the AOB study across time will include those relevant to population health such as obesity, injuries, recreation, chronic/inflammatory disease, and developmental disorders. Planned domains for a 5 and 8 year follow-up also include recreation, screen time, sleep, and oral health, among others. Detailed in-home anthropometric and developmental assessments, as well as DNA collections are also planned for in subsequent follow-up data collections.

## Results

### Characteristics of the AOB participants

Participant demographics, pregnancy characteristics, and labour and birth outcomes are presented in Table 2 and Table 3. Psychosocial characteristics during the prenatal and postpartum period are shown in Table 4. The majority of participants were less than 35 years of age at delivery (76%), Caucasian (79%), and Canadian born (78%). Almost all were either married or living in a common-law relationship (94%). Eighty-nine percent had completed at least some post secondary education and 69% reported an annual household income greater than $80,000. The index pregnancy was the first for approximately 1/3 of the sample and almost half were nulliparous, which suggests that a significant proportion of these first-time mothers had experienced fetal loss in previous pregnancies (27%). The majority had been trying to become pregnant (80%) and most reported feeling happy about being pregnant (87%). Approximately 3% conceived through assisted reproductive technologies, including fertility-enhancing drugs, artificial insemination, and in-vitro fertilization. Forty-one percent gained weight that aligned with the recommended guidelines for gestational weight gain based on pre-pregnancy body mass index [21], and one-quarter delivered by caesarean-section. The study yielded 36 sets of twin births. The overall preterm birth rate was 7.9%. Among singleton births, the preterm birth and Small for Gestational Age (SGA) rates were 7.3% and 10.6%, respectively. Almost all mothers initiated breastfeeding, if only for a short time (98%); of these, 61% were exclusively breastfeeding at 4 months postpartum.

**Table 2:** Demographic characteristics of the AOB study participants

Characteristic	n(%)
**Maternal age at delivery (n=2670)**	
19-24	153 (5.7)
25-29	716 (26.8)
30-34	1156 (43.3)
35-39	553 (20.7)
40+	92 (3.4)
**Marital status (n=3354)**	
Married/Common Law	3165 (94.4)
Other	189 (5.6)
**Education (n=3356)**	
High school or less	370 (11)
Some or completed university/college	2458 (73.2)
Some or completed grad school	528 (15.7)
**Ethnicity (n=3354)**	
Caucasian	2636 (78.6)
Non-Caucasian	718 (21.4)
**Income (n=3252)**	
< $40,000	299 (9.2)
$40,000 - $79,000	717 (22)
≥$80,000	2236 (68.8)
**Born in Canada (n=3360)**	
Yes	2623 (78.1)
No	737 (21.9)

**Table 3:** Pregnancy and labour/delivery characteristics of the AOB study participants

Characteristic	n(%)
**Pregnancy intention (n=3355)**	
Trying to get pregnant	2698 (80.4)
Not trying to get pregnant	657 (19.6)
**Feelings about pregnancy (n=3348)**	
Happy	2913 (87)
Unhappy/not sure	435 (13)
**Gravidity (n=3338)**	
Nulligravida	1192 (35.7)
Primi/Multigravida	2141 (64.3)
**Parity (n=3340)**	
Nulliparous	1637 (49)
Primi/Multiparous	1703 (51)
**Weight gain during pregnancy^a^ (n=3002)**	
Inadequate	895 (29.8)
Adequate	1239 (41.3)
Excessive	868 (28.9)
**Method of delivery (n=3055)**	
Vaginal	2297 (75.2)
Caesarean section	758 (24.8)
**Gestational age (n=3032)**	
<34wks	51 (1.7)
34-36 wks	190 (6.2)
37+ wks	2791 (92.1)
**Small for Gestational Age (singletons; n=2836)**	
SGA	300 (10.6)
Not SGA	2536 (89.4)
**Large for Gestational Age (singletons; n=2836)**	
LGA	251 (8.9)
Not LGA	2585 (91.1)
**Breastfeeding initiation (n=3057)**	
Yes	2993 (97.9)
No	64 (2.1)
**Exclusive breastfeeding at 1 wk (n=2969)^b^**	
Yes	1786 (60.2)
No	1183 (39.8)
**Exclusive breastfeeding at 4-months (n=2976)^b^**	
Yes	1809 (60.8)
No	1167 (39.2)

^a^ Difference in weight between 34-36wks and pre-pregnancy

^b^ Among those who initiated breastfeeding

**Table 4:** Psychosocial characteristics of the AOB study participants

Characteristic	n(%)
**Prenatal**	
Depression, EPDS^a^ ≥13 (n=3384)	
Yes	405 (12)
No	2979 (88)
Anxiety, SAI^b^≥ 40 (n=3363)	
Yes	924 (27.5)
No	2439 (72.5)
Stress, PSS^c^ 80^th^ percentile (n=3376)	
Yes	1041 (30.8)
No	2335 (69.2)
Social support, MOS^d^ total ≤ 69 (n=3379)	
Inadequate	645 (19.1)
Adequate	2734 (80.9)
Optimism, LOT-R^e^ 20^th^ percentile (n=2925)	
Low optimism	582 (19.9)
High optimism	2343 (80.1)
	
**4 months postpartum**	
Depression, EPDS^a^≥13 (n=3041)	
Yes	152 (5)
No	2889 (95)
Anxiety, SAI^b^ ≥ 40 (n=2942)	
Yes	440 (15)
No	2502 (85)
Stress, PSS^c^ 80^th^ percentile (n=3004)	
Yes	714 (23.8)
No	2290 (76.2)
Social support, MOS^d^ total ≤ 69 (n=3012)	
Inadequate	412 (13.7)
Adequate	2600 (86.3)
Parenting Morale Index, PMI^f^ 20^th^ percentile (n=2931)	
Low parenting morale	491 (16.8)
High parenting morale	2440 (83.2)

^a^ Edinburgh Postnatal Depression Scale (EPDS) [40]

^b^ State-Trait Anxiety Inventory (state anxiety scale; SAI) [41]

^c^ Perceived Stress Scale (PSS) [42]

^d^ Medical Outcomes Study (MOS) Social Support Scale [38]

^e^ Lifetime Orientation Test-Revised (LOT-R) [43]

^f^ Parenting Morale Index (PMI) [44]

Psychosocial characteristics in the AOB cohort were assessed using standardized tools (see additional file 1). Prenatal psychosocial health was operationalized as scoring in the excessive symptom range (high or low depending on the construct) at one or both of the prenatal data collection time points. Women in the AOB cohort reported prevalences of prenatal depression, anxiety, and stress of 12%, 28%, and 31%, respectively. At 4 months postpartum, the rates were lower, at 5% for depression, 15% for anxiety, and 24% for stress. Perceived social support remained high at both time points (>80%) and the majority of women reported high optimism (80%) and parenting morale (83%) (Table 4).

### Characteristics of discontinued participants

In order to gain a better understanding of the variables that may be associated with study attrition, which would inform the extent of possible selection bias, we compared the demographic characteristics between those women who dropped out of the study after the first questionnaire, excluding pregnancy losses, and those who continued to the second and/or third data collection (Table 5). Results in Table 5 show that women who stopped participation after the first questionnaire for reasons other than pregnancy loss were more likely to be younger, non-Caucasian and foreign born, and to report lower education and household income levels. Compared to those who continued, discontinuers were less likely to be married or living in a common-law relationship, and reported poorer psychosocial health in early pregnancy (Table 5). There were no significant differences between the two groups in terms of gravidity, or feelings about pregnancy. We were unable to carry out an assessment of characteristics of women who agreed to participate but then failed to return a questionnaire because ethically we were unable to collect any information about data about these women at recruitment.

**Table 5:** Comparison between AOB discontinuers^a^ and AOB continuers^b^

Characteristic	Drop-outs (n=123)	Continuers (n=3208)	p-value
Maternal age			
<30 years	56 (49.1)	1094 (36.5)	0.006
30+ years	58 (50.9)	1904 (63.5)	
Marital status			
Married/common-law	102 (83.6)	3030 (94.8)	<0.001
Other	20 (16.4)	166 (5.2)	
Education			
High school or less	32 (26.0)	336 (10.5)	<0.001
More than high school	91 (74.0)	2861 (89.5)	
Ethnicity			
Caucasian	85 (69.7)	671 (21.0)	0.014
Non-Caucasian	37 (30.3)	2525 (79.0)	
Income			
<$40K	29 (24.4)	266 (8.6)	<0.001
$40+K	90 (75.6)	2833 (91.4)	
Gravidity			
Nulligravida	43 (35.0)	1138 (35.8)	0.85
Primi/Multigravida	80 (65.0)	2041 (64.2)	
Born in Canada			
Yes	87 (70.7)	2508 (78.4)	0.045
No	36 (29.3)	693 (21.6)	
Depression in early pregnancy			
Yes	27 (22.1)	239 (7.5)	<0.001
No	95 (77.9)	2952 (92.5)	
Anxiety in early pregnancy			
Yes	37 (32.2)	506 (16.3)	<0.001
No	78 (67.8)	2589 (83.7)	
Stress in early pregnancy			
Yes	50 (41.3)	660 (20.9)	<0.001
No	71 (58.7)	2505 (79.1)	
Social support in early pregnancy			
Inadequate	25 (20.8)	412 (13.0)	0.013
Adequate	95 (79.2)	2762 (87.0)	
Feelings about pregnancy			
Happy	100 (81.3)	2783 (87.2)	0.057
Unhappy/not sure	23 (18.7)	409 (12.8)	

### Comparison to the target population

We compared the demographic and pregnancy characteristics, as well as the delivery and postpartum experiences of the AOB study participants to provincial and national statistics drawn from the Maternity Experiences Survey (MES) [22,23]. Using post-census (2006 Canadian Census) data, the MES is a cross-sectional sample survey that serves as the target population of women and families who become parents in Canada. As the MES was restricted to women with singleton births, we invoked this criterion for the AOB sample to facilitate comparisons. A greater proportion of women in the AOB sample were older (≥35 years) and reported a higher household income compared to MES participants (Table 6). In terms of pregnancy characteristics, women in the AOB sample were more likely to have received a first ultrasound before 18 weeks gestational age and to have attended prenatal or childbirth education classes. Percentages for the remaining demographic and pregnancy characteristics were, in general, similar between AOB and MES participants. The preterm birth rate (singletons) for AOB was higher than that reported in the MES, and AOB participants reported a shorter length of stay for both vaginal and caesarean-section deliveries. Compared to MES participants, AOB participants were less likely to report their physical postpartum health as very good or excellent, yet were less likely to score 13 or above on a widely used postpartum depression scale (Table 6). On average, the remaining pregnancy and postpartum characteristics compared between the two samples were similar.

**Table 6:** Comparison of AOB participants to MES^a^ participants

Characteristic	AOB %	Alberta %	Canada %
Demographic characteristics			
≥35 years	24.1	15.6	17.5
Postsecondary completed	76.3	69.5	72.1
>$40K	92.3	77.8	72.6
Primiparous^b^	48.9	46.0	44.7
Pre-pregnancy BMI (mean)	24.3	24.4	24.4
Pregnancy characteristics^c^			
Number of prenatal care visits (mean)	12.8	13.0	12.9
Gestational age at first prenatal care visit (mean)	9.1	7.2	7.5
Initiated prenatal care in first trimester (<14 weeks)	93.1	94.9	94.9
First ultrasound <18weeks	85.6	63.4	66.8
Attended prenatal or childbirth education classes	41.2	33.4	32.7
Satisfied with timing of pregnancy	52.6	50.9	49.5
Feeling happy^d^ upon realization of pregnancy	87.0	90.8	93.0
Intended to breastfeed	96.2	93.8	90.0
Delivery and postpartum experiences			
Preterm birth rate	7.3	6.3	6.2
Caesarean section delivery	24.5	27.3	26.3
Short length of maternal stay in hospital			
Vaginal (<2 days)	66.8	60.7	33.6
Caesarean section (<4 days)	79.9	59.1	53.0
Initiated breastfeeding	97.8	94.6	90.3
Scoring ≥13 on Edinburgh Postnatal Depression Scale	5.1	6.5	7.5
Rated postpartum health as very good or excellent	53.9	73.6	72.5
Postpartum BMI (mean)	25.6	25.5	25.4

^a^ Maternity Experiences Survey 2006-2007; comparisons involve singletons only

^b^ according to status at birth

^c^ assessed during postpartum in MES (retrospective recall); assessed during pregnancy in AOB

^d^ “happy” derived from collapsing responses of “somewhat happy” and “very happy”

Although the MES may be a less than ideal comparison for representativeness, given that AOB and MES employ different sampling strategies (i.e., stratified sampling in MES, non-stratified sampling in AOB), the range of factors assessed in the MES allows for a wide range of comparisons, beyond sociodemographic characteristics and birth indicators. Further comparisons with other data sources at the local and provincial level such as administrative data on perinatal health and Census community profiles during or close to the study time period suggest that the AOB participants are generally representative of the pregnancy and parenting population at the local (city) and provincial levels. For example, the average age of women in Calgary and Alberta giving birth in 2010 was 30.8 and 29.5 years [24]. In the AOB study, the average age at delivery was 31.2 (SD=4.4). Approximately one-quarter of women in Calgary were foreign-born and one-quarter were a visible minority according to the Canadian Census [25], with similar percentages seen in the AOB study (Table 2). Furthermore, 53% of women in the AOB study report a household income of over 100K, which aligns with the median income of couple families according to recent statistics from Statistics Canada for 2010 (approximately 97K) [26].

### Comparison to perinatal surveillance data

Recent data on perinatal indicators [27] report a singleton preterm birth rate of 7.9% and 8.8% in Canada and Alberta, respectively. The AOB preterm birth rate for singletons of 7.3% falls below both the provincial and national rates; on the other hand, the AOB SGA rate of 10.6% is greater than the corresponding provincial and national rates. Taken together, this suggests possible misclassification of both birth weight and gestational age data according to self-report. Validation work with medical charts for important labour and delivery outcomes has been completed and is described elsewhere in this issue [28]. Although relatively high agreement was found between the two data sources for select perinatal indicators [28], misclassification cannot be ruled out when comparing study rates to perinatal surveillance data. Finally, mothers in the AOB cohort had much higher breastfeeding initiation rates than those reported for both Canada and Alberta (98% vs. 87% and 91%, respectively).

## Conclusion

### Significance

Emerging evidence recognizes the importance of prenatal and early life events on the long term development of children [29,30]. The AOB cohort has the unique opportunity to inform complex questions about the relationship between biology, early experiences, and developmental outcomes, and to contribute to a better understanding of the current circumstances of importance to families for stakeholders, policy and decision makers. An informed picture of the early determinants of childhood development and family outcomes is potentially important for not only prevention of disability and ill-health but also in developing an understanding of mechanisms underlying associations between early and later life outcomes (e.g., early socioeconomic status (SES) as a predictor of childhood intelligence and its role in explaining the association between childhood intelligence and risk for adult disease; [31]). Future studies examining associations between risk factors and later life outcomes must ensure adequate control for potential confounders. Such early life determinants of such risk factors, that are outcomes in themselves, require elucidation and adequate measurement. A key advantage of the AOB cohort, like some other established longitudinal cohorts (e.g., ALSPAC, Generation-R), is that its prospective data collection began in pregnancy. Although birth cohorts and cohorts that begin in early childhood are important sources for life course research, pregnancy cohorts are well positioned to overcome methodological limitations such as recall bias for exposures and confounding variables in pregnancy. Common to all cohort studies, sample attrition over time may be a source of selection bias for the AOB cohort (see below). Although the AOB cohort demonstrated a retention rate of 90% of participants between the first and third questionnaire, there was an 86% response rate for the 12 month data collection. Although this latter rate is still high, the decrease across time serves as a reminder that intensive participant engagement is an important component for ongoing cohort maintenance and follow-up.

Tracking typical and atypical trajectories of child development as well as risk factors and effect modifiers is important for the development of preventative strategies. We have incorporated assessment tools to screen for atypical development as part of the 12, 24, and 36 month follow-up data collections. For example, the MacAurthur-Bates Communicative Development Inventories [32] are included during follow-up to identify those children at risk for language delay. To our knowledge, no previous population-based cohort exists of this size that incorporates three assessments of atypical child development coupled with rich maternal data and other gold standard tools. Follow-up data collections will also allow for examining typical and atypical trajectories of maternal and family well-being after the birth of a new baby. Longitudinal data analyses will be performed to examine precursors and outcomes of trajectories. We will also track outcomes as part of surveillance undertaking for the AOB cohort. Some specific projects that will use longitudinal data include: examining early risk factors for language delay; intergenerational transmission of psychosocial risk; and long-term outcomes for late-preterm infants and their families.

### Threats to validity

A main source of potential bias for longitudinal studies is that due to non-response; pregnancy and birth cohorts are no exception. Non-response can affect both external and internal validity. In general, non-response can take three forms: unit non-response, or absence of the target sample at study outset; temporary or wave non-response; and permanent non-response, commonly referred to as attrition [33]. An analysis of unit non-response generally comprises a comparison of the study population to the eligible or target population, and may derive from previous collection of minimal data sets on individuals who either refused to participate or were missed [34], or the use of administrative data sources with total population coverage of births or pregnancies [14,18]. Temporary and permanent non-response can be assessed if baseline information is collected before drop-out; our comparison between continuers and discontinuers is an example of an assessment of this type of non-response and threat to validity. In line with other cohort studies, non-continuers in the AOB were more likely to report poorer mental health and lower socioeconomic status [35-37]. We will continue to examine the characteristics of discontinuers across time as selection bias due to attrition may become an increasing threat to validity, in particular when examining lifecourse associations. In the AOB cohort, other potential sources of bias such as information bias (e.g., misclassification bias, recall bias) and bias due to confounding are kept to a minimum due to the prospective nature of data collection, use of standardized tools, and assessment across a range of variables including different data sources. However, we cannot discount the possibility that reporting bias due self-report will remain a potential threat to validity, and, where possible, we will utilize medical records and administrative sources of information and/or conduct validation analyses between different data sources to maintain internal validity. Although vulnerable women may be at higher risk of discontinuation, variability in ethnicity, SES etc. is present, and tends to reflect the urban Calgary parenting population, which allows for examining associations for these factors, maintaining internal validity at the expense of external validity (generalizability).

### Summary

The AOB cohort, in general, is representative of the pregnant and parenting population in a Canadian urban setting, Important research and policy questions are currently under examination, results which have the potential to add to the evidence base and inform decision makers about the health and well-being of pregnant women and their families. The AOB cohort will continue to be a significant Alberta resource that will have implications far beyond its local roots.

## List of abbreviations used

AOB: All Our Babies; ALSPAC: Avon Longitudinal Study of Parents and Children; SES: Socioeconomic Status; MES: Maternity Experiences Survey

## Competing interests

The authors declare that they have no competing interests.

## Authors’ contributions

SCT is responsible for the overall integrity, progress and timely completion of the AOB study. AWL is responsible for all lab-based queries. AWL, KMB, DAM, SJL, SMD, CEP, ADB, and SCT participated in the design of the study. SWM drafted the manuscript, performed data linkage, and conducted all statistical analyses. All authors have read and approved the final manuscript.

## Supplementary Material

Additional file 1 Variables assessed by questionnaire in the AOB study by data collection time point Click here for file

## Declarations

This article has been published as part of *BMC Pregnancy and Childbirth* Volume 13 Supplement 1, 2013: Preterm Birth: Interdisciplinary Research from the Preterm Birth and Healthy Outcomes Team (PreHOT). The full contents of the supplement are available online at http://www.biomedcentral.com/bmcpregnancychildbirth/supplements/13/S1.

All of the publication fees will be funded by the Preterm Birth and Healthy Outcomes Team Interdisciplinary Team Grant (#200700595) from Alberta Innovates - Health Solutions, formerly the Alberta Heritage Foundation for Medical Research.
